# The cost of firearm violent crime in British Columbia, Canada

**DOI:** 10.3389/fpubh.2022.938091

**Published:** 2023-01-13

**Authors:** Fahra Rajabali, Kate Turcotte, Alex Zheng, Nick Pauls, Tony Nguyen, Evelyn Kalman, Vedrana Covic, Ian Pike

**Affiliations:** ^1^BC Injury Research and Prevention Unit, BC Children's Hospital, Vancouver, BC, Canada; ^2^Public Safety and Police Services Unit, Policing and Security Branch, Ministry of Public Safety and Solicitor General, Victoria, BC, Canada; ^3^Department of Pediatrics, University of British Columbia, Vancouver, BC, Canada

**Keywords:** firearm injury, costs, criminal justice system costs, health care costs, violent crime

## Abstract

**Introduction:**

This study aimed to quantify the total cost of violent firearm-related offenses in British Columbia in 2016 Canadian dollars over a five-year period, 2012 to 2016. The purposes of this study were to estimate the direct costs to the health care system and indirect costs to society for violent firearm injuries and deaths; and to estimate criminal justice system costs pertaining to firearm incidents.

**Methods:**

Human and economic costs to the health care system and productivity losses were calculated using health administrative datasets such as B.C. Vital Statistics and Discharge Abstract Database. Criminal justice system costs pertaining to firearm incidents were estimated by applying weighted average costs to aggregate expenditures using methodology consistent with that used by Statistics Canada.

**Results:**

There was a total of 108 deaths and 245 hospitalizations resulting from violent firearm injuries. The total estimated cost of all violent firearm crime averaged $294,378,985 per year; human costs averaged $188,416,841 per year, where health care costs averaged $3,910,317 per year, productivity losses from workforce and household averaged $17,299,054 and $4,559,470 per year, respectively, and loss of life averaged $162,648,000; and $105,021,145 in criminal justice system costs, and $941,000 in programming costs.

**Conclusion:**

This study clearly demonstrates the significant cost of violent firearm injury in British Columbia and the impacts on the health care system, criminal justice system, and to society at large, particularly within the criminal justice system where the costs were significantly higher than health care.

## Introduction

Firearms are a lethal means to homicide, suicide, and unintentional injuries, highlighting an important worldwide public health issue with social and economic costs that extend beyond life lost. Firearm injuries are a major concern in the United States (US), with assaults being the most common overall mechanism (1). Canada ranks 9 out of 36 peer Organization for Economic Co-operation and Development countries in firearm mortality rates (2). Nearly one-in-three homicides in Canada are firearm-related, reflecting a significant uptrend where shootings have consistently exceeded stabbings and beatings as the most common method of homicide (3, 4). The rate of firearm-related crime in Canada increased from 2018 to 2019 by 7% (5). Ontario, one of the major provinces in Canada, has estimated the occurrence of firearm-related injury to one per day (6). Firearm-related crime comprises a very small proportion (0.5%) of all violent crime in British Columbia (B.C.) (4), but is responsible for a disproportionate level of harm to individuals, families, and communities. Improved public safety for all British Columbians is one of the objectives of the government (7). To understand and identify the magnitude of investments toward public safety and crime prevention, it is important to quantify the dollar value and social impact of firearm violent crime.

The impact of firearm violent crime can be wide reaching and long lasting, requiring considerable societal resources to address the problem. Estimating the cost of firearm incidents is complicated and challenging, with the outcomes and consequences varying widely from case to case. Individuals, families, and communities bear hefty costs due to firearm injury, and these incidents have the potential to devastate lives, to incite fear, and to impose heavy social, psychological, and financial burden (8). In addition, health care costs such as physician, procedure, facility, radiology, and medication costs from firearm injury have shown to increase 3 to 20 times six-months post injury, indicating greater post injury care needs for physical therapy and rehabilitation (9). The highest rates of firearm homicide occur in the US (10), hence the many US-based studies that have quantified the financial burden of the treatment of firearm deaths (11), and injuries in hospitals and emergency departments (ED) (12–15). However, firearms control and firearms-related incidences in Canada is unlike the US. In Canada, one national study estimated the police cost per firearm incident for 8,885 police-reported incidents at $23,609, and the cost per victim for 9,469 firearm victims at $287,000 in 2008 (16). These estimates included personal costs and intangible costs, as well as third-party costs such as loss of affection/enjoyment to family members, social service operating costs, as well as costs to other persons harmed or threatened (16).

To our knowledge, this study is the first to estimate the total cost of violent firearm injury in B.C. This study aimed to quantify the total cost of violent firearm-related offenses in B.C., with the objectives to: i) estimate the direct human costs to the health care system (treatment and care), as well as indirect costs to society (productivity losses) for violent firearm injuries and deaths; and, ii) estimate criminal justice system costs related to firearm incidents. This study was undertaken with a societal, or population, perspective. It included costs that have an impact on society as a whole, including individuals, families, employers, and the government. The application of a societal perspective supports a public health viewpoint and supports policies aimed at minimizing losses or maximizing gains to society.

## Methods

Firearm incidents, including those that result in injury or death, are reported by B.C. police forces as crimes against the person. Data for the five-year period January 1, 2012 to December 31, 2016, inclusive, were used to determine firearm-related incident information, associated injuries and deaths, and population statistics. These years were selected as 2016 was the most recent year with available hospitalization data at the time of this study, and five years of data were aggregated to allow for adequate case counts, and to “smooth” higher- and lower-than-average annual counts. Estimated average costs across the five-year period were applied to calculate costs in 2016 Canadian (CAN) dollars. All datasets used to calculate costs were not linked and therefore the incidents were not mutually exclusive and chances of double counting remain.

**Figure 1:**
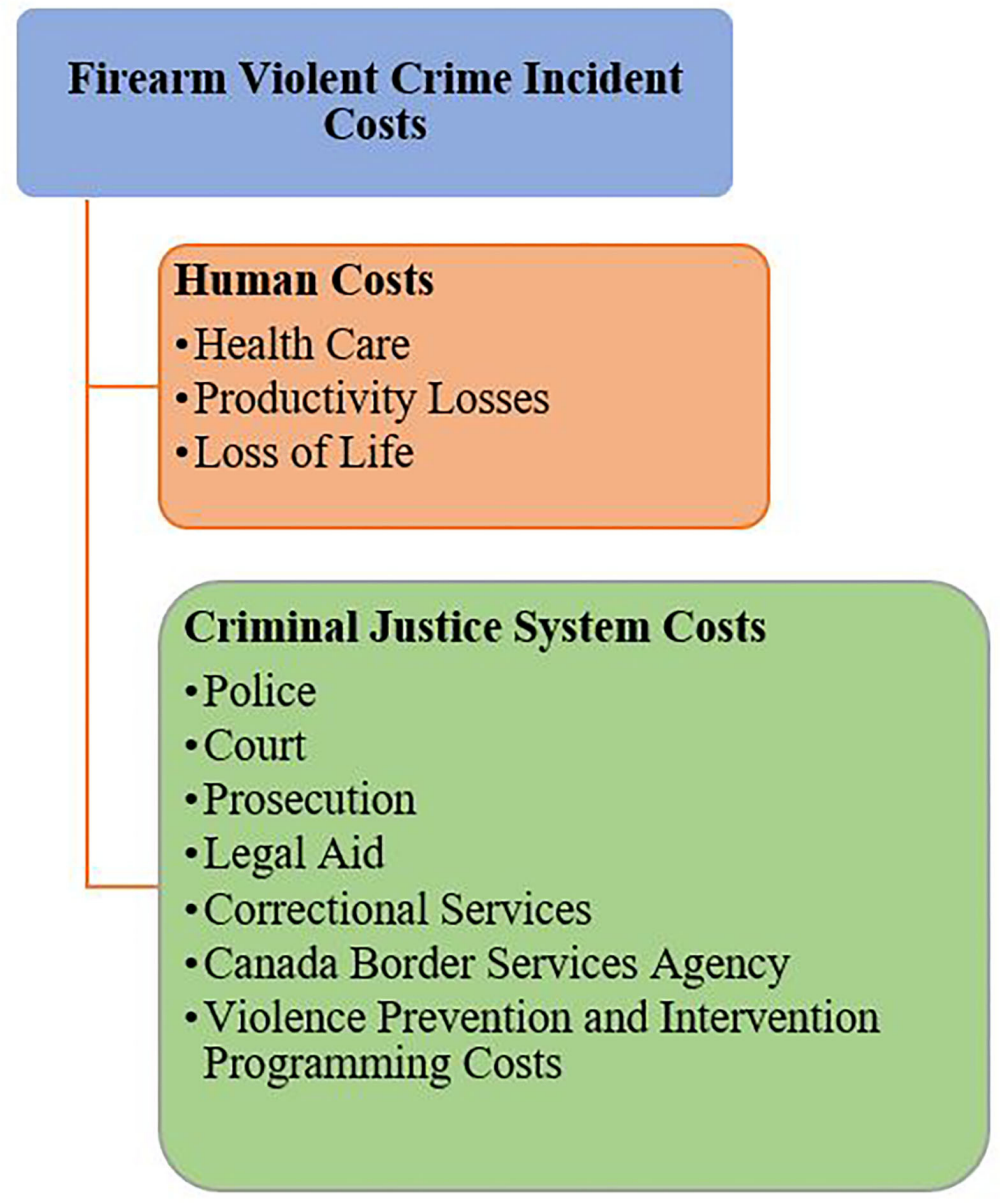
Categorization of firearm violent crime incident costs.

The categorization of firearm violent crime incident costs is illustrated in Figure 1.

### Human costs

Human costs were calculated using an incidence costing, human capital approach covering the lifetime of the injured individual. Human costs were primarily calculated using the ERAT—Electronic Resource Allocation Tool (17), a spreadsheet tool with the purpose to calculate the incidence costs of injury, which provides a classification and costing framework based on existing injury data and data available from the published injury costing literature (18) and/or credible sources, such as Statistics Canada and the Canadian Institute for Health Information. Data sources used to calculate human costs are listed in Table 1.

**Table 1:** Data sources for human costs.

**Cost category**	**Details**	**Data source for cost information**
Death	Deaths that occurred at the scene, ambulance transportation, treatment in ED and hospital prior to death, coroner and autopsy services, and funeral services	B.C. Vital Statistics Agency, B.C. Center for Disease Control, Chronic Disease and Injury data mart
Hospitalization	Ambulance transportation, physician and specialist services, hospital costs, and long-term medical and rehabilitation costs	Discharge Abstract Database (DAD), Ministry of Health
Emergency Department (ED) visits	Ambulance transportation, physician and specialist services, treatment in ED, and long-term medical and rehabilitation costs	Ratios derived from DAD and the BC National Ambulatory Care Reporting System (NACRS)
Disability	Short term (within the year of injury) and long-term (12 months to lifetime). Employed with disability for permanent and total disability	Disability weights based on patient-reported data from a multinational injury cohort (19) Statistics Canada, Canadian Survey on Disability
Out-of-hospital treatment	Ratios of episodes and related costs of non-hospitalized to hospitalized cases	Databook on Non-fatal Injury Incidence, Costs and Consequences (18)
Coroner or medical examiner services and autopsy costs		Ministry of Solicitor General
Ambulance transportation costs		B.C. Emergency Health Services
Physician care expenditures		Canadian Institute for Health Information. National Physician Database—Utilization Data
Cost of standard hospital stay		Canadian Institute for Health Information
Funeral costs		Life Insurance Canada
Informal and formal caregiving		Public Health Agency of Canada (20)
Average earnings, B.C. participation rate and unemployment rate		Statistics Canada (21)

Direct (health care) costs comprise all goods and services used for the diagnosis, treatment, and care of people experiencing injury. Indirect costs (productivity losses), derived using the human capital approach, represent the injured individual's inability to perform their normal activities of daily living and are defined as lost resources resulting from inability to work, premature death, and costs of informal care (22). For deaths, this is the forgone future income, and for morbidity, these are time away from work and years of disability resulting in forgone income, measured for permanent partial and permanent total disability. Permanent partial disability is one “from which partial recovery is anticipated, along with a return to some form of employment. Complete loss of earning power is expected prior to recovery, after which the worker is expected to return to employment with wages below preinjury wages” (18). Permanent total disability is “a condition equivalent to complete and permanent loss of earning power” (18). This approach was taken with the assumption that permanent total and partial disabilities apply for both work-related and other injuries (23).

Indirect costs are affected by age, the base year wage rate, the probability of participating in the labor market, and having participated, the probability of being employed, the real wage growth rate, and the discount rate. The value of time lost from work due to morbidity, disability, and premature death was measured using B.C. earnings data. Both direct and indirect costs were discounted to a present value in 2016 at 1.5% per annum (24). The effective annual discount rate affects all costs beyond the 1st year. These include all indirect costs associated with the loss of income, excluding the indirect costs of a hospital stay, which are assumed to occur in the current year, and includes the long-term direct costs associated with disability. Indirect costs reflect lost earning, discounted and adjusted for labor market participation and unemployment, of all future income over the relevant period within the working life of an individual from ages 15 to 64 years, inclusive. A real wage growth rate of 1% per year was assumed (17). The annual real wage growth rate affects future wage levels for the indirect costs.

Death and hospitalization data were extracted from the BC Vital Statistics and the Discharge Abstract Database (DAD), respectively (Table 1), using the 10th revision of the International Statistical Classification of Diseases and Related Health Problems, Canadian Adaptation (ICD-10 CA), codes for homicide, injuries inflicted by another person with intent to injure or kill, those due to legal intervention, and those that are undetermined. The codes included were, X93–X95: assault by handgun, rifle, shotgun, and larger firearm discharge, as well as other and unspecified discharge; Y22–Y24: handgun, rifle, shotgun, and larger firearm discharge, as well as other and unspecified discharge with undetermined intent; Y35: legal intervention involving firearm discharge (with machine gun, revolver, fire pellet, or rubber bullet). In-hospital deaths were excluded from the total hospital count and included in the calculation of the costs of firearm deaths. Similarly, ED costs were excluded from the hospitalization costs to avoid double counting. The BC National Ambulatory Care Reporting System (NACRS) data, source for ED visit data, do not include the external cause nor mechanism of injury, and are underreported and incomplete. To obtain an estimate of the ED visits, a ratio of ED visits to hospitalizations for firearm injury was applied using the ratio from the health region that had all its hospitals reporting to NACRS as a reference (25). US ratios were used to calculate direct morbidity costs for out-of-hospital episodes and related costs of non-hospitalized to hospitalized cases, which were assumed to be similar for B.C. (18).

The incidence of deaths, hospitalizations, ED visits, and disability; values for population and length of stay; resource intensity weights for resources allocated to the hospital stay; the average cost of hospital stay; and the unemployment rate, participation rate, and average weekly earnings were all entered into ERAT to obtain the corresponding direct and indirect costs. As the physician costs and ambulance costs for hospitalizations and ED visits were not included, they were appended to the hospitalization and ED costs derived from ERAT.

Direct death costs were estimated on a complete episode of events due to a firearm death. These events range from costs incurred as a result of deaths occurring at the scene, ambulance transportation costs, treatment occurring in the ED and hospital prior to the death, coroner and autopsy costs, and funeral costs. As ERAT only included indirect death costs, total death costs were calculated by appending direct death costs to the indirect death costs from ERAT.

Unpaid labor includes caregiving, volunteer work, household activities, or any other activity that occurs outside of the standard labor market. ERAT only included estimates of production losses of paid work as a result of morbidity and premature death. The value of lost production was estimated for the working-age population comprising individuals aged 15–64 years. For this study, it was assumed that all individuals contribute to society. Caregiving, defined as care received by the injured individual within the year of injury and the main condition for which the injured individual sought help, can be classified as either a direct cost or an indirect cost depending on whether a formal, or direct, payment was made (20). Informal caregiving provided by family, friends, and neighbors are considered indirect costs, and formal caregiving provided by paid workers and organizations are considered direct costs. The 0.02% of formal and 0.47% of informal caregiving for injury was applied to the direct and indirect costs (20). Primarily based on Canada's 2009 General Social Survey, Zhang and Qin found that the ratio of household work lost to wage work lost by victims of violence was 0.35855 for women and 0.24472 for men (16). For gunshot victims under age 65, those ratios were applied to the earnings losses to estimate the household work losses. Gunshot victims over age 65 were assumed to lose the same amount of household work per year as younger victims.

There is significant quality of life lost due to deaths, or a Value of a Statistical Life (VSL), the additional cost that individuals would be willing to bear for improvements in health and safety. As recommended by the Government of Canada, VSL was calculated for violent firearm deaths by applying an average VSL value of $6.5 million in 2007 Canadian dollars, converted using the Statistics Canada consumer price index (26, 27), which is equivalent to $7.53 million in 2016 Canadian dollars. It should be noted that the VSL is not fixed, as individuals' risk-money trade-offs vary across the population and over time as age and economic circumstances of the individual changes.

Vision care and prescription drug expenditures outside of hospital care were excluded as data were not available. Previous studies included personal costs, such as economic dependence and social isolation; intangible costs, such as pain and suffering; and third-party costs such as loss of affection to, or enjoyment of, family members, as well as social services operating costs (16). The current study did not include these costs due to a lack of available data. These study methods therefore represent a conservative approach and are considered to be an underestimate of the true costs related to violent firearm incidents in B.C.

### Criminal justice system costs

Criminal justice system costs were estimated by applying weighted average costs to aggregate expenditures using methodology consistent with that used by Zhang and Qin and Statistics Canada (16, 28). Data calculations for criminal justice system costs are outlined in Table 2 (descriptions and data sources available in Supplementary material). Further information on the methodology applied can be obtained from Zhang and Qin (16) or Statistics Canada (28).

**Table 2:** Data calculations for criminal justice system costs.

**Cost category**	**Calculation**
Police (29–31)	1. Total Severity of Offense = Number of Total Incidents x Severity Weight 2. Weighted Proportion of Severity = Total Severity of Offense/Sum of All Total Severity Weight of Offenses 3. Overall Police Expenditure = Weighted Proportion of Severity x Police Expenditures Spent on Crime Related Activities 4. Per Incident Police Costs = Overall Police Expenditure/Number of Total Incidents 5. Police Costs for Firearms = Per Incident Police Costs x Number of Firearms Related Incidents
Court services	1. Average court case cost = Total provincial court expenditures per year/total number of criminal court cases concluded + total number of active civil court cases for the same period 2. Total court services costs = Average court case cost x total number of firearms-related court cases
Prosecution (32)	1. Average prosecution cost per case = Total expenditure for prosecution services/ total number of criminal cases (adult and youth) by year 2. Total prosecution costs = Average prosecution cost per case x total number of firearms-related offense cases processed in criminal court
Legal aid (33)	1. Legal Aid cost per case = Total legal service expenditures/Number of total criminal cases 2. Total legal aid costs = Legal Aid cost per case x total number of firearm offenses
Federal custody	1. Total federal incarceration costs for offenders + total cost of supervising offenders on parole 2. Total federal custody costs for crimes against the person + non-murder and violations causing death + aggravated assault
Provincial custody (34)	1. Provincial incarceration costs = Average daily provincial/territorial incarceration cost/person x total number of provincial incarceration days for offenders 2. Provincial parole costs = Average daily provincial parole cost x number of provincial parole days 3. Total provincial custody costs = Total provincial incarceration cost + total provincial parole costs
Conditional sentence (35)	1. Average daily cost = Average daily provincial incarceration cost/person x 0.20 2. Total conditional sentence cost = Number of offenders receiving a conditional sentence x average length in days of conditional sentence x average daily cost for conditional sentence per offender
Probation costs	1. Total probation length in number of days = Number of offenders receiving probation x average probation length in number of days for offenders 2. Total probation costs = Average daily probation cost/person x probation length in number of days for offenders
Fines	Total fines = Average value of a fine for offenders x number of offenders receiving a fine as a sentence for firearm offenses
Canadian Border Services Agency criminal investigation (36, 37)	1. Average CBSA criminal investigation cost per case = Total CBSA national criminal investigations expenditures/ the total number of CBSA criminal investigation cases 2. Total CBSA costs = Average cost per case x total number of CBSA prosecutions and seizures involving firearms estimated for the Pacific region

Police and court costs were calculated using aggregate costs available from federal government official sources (Table 2, Supplementary material), however, the cost per crime was not always available. The cost also varied considerably according to the seriousness of each incident. This difficulty also applied when estimating the costs of prosecution and legal aid. While the aggregate expenditures on criminal matters were available, there was no information to indicate the actual number of cases of firearm-related crimes that involved prosecution and/or legal aid, or the associated cost per case.

In estimating the police costs for firearm incidents reported to police as crimes against the person, total costs of police expenditures were assigned to the various crimes according to their severity weights using methodology developed by Statistics Canada (28), with serious crimes assigned higher weights (Table 2, Supplementary material). Average costs per case were estimated for court, prosecution, and legal aid. With respect to correctional service costs, the average daily cost of provincial and federal incarceration was applied, and this average cost did not vary dramatically by offense type (Table 2, Supplementary material).

Violence prevention and intervention programming costs were internally retrieved from the B.C. Ministry of Public Safety and Solicitor General.

For the purposes of this study, violent crime included all person-related offenses processed within the judicial system that may or may not have resulted in injury. Incidents classified as undetermined were included with violent crime.

Human, criminal, and justice costs, and violence prevention and intervention programming costs were summed to obtain the total firearm violent crime costs.

### Data limitations

The data sources used in this study are largely reliant on existing administrative data, where the quality of the data is reliant on the expertise of professional data coders and thus may exhibit inherent biases and inaccuracies. Injuries treated in doctor's offices or walk-in clinics are not accounted for as data were unavailable. There is a large data gap for hospitalized injuries requiring ongoing care outside of the hospital setting, ranging from short periods to long-term permanent disability.

Unpaid labor was not included, such as volunteer work, or any other activity that is outside of the standard labor market. Indirect costs did not include transfer payments made by government or social services, as they are a reallocation of resources and the net effect of the transfer to society is zero. Furthermore, indirect costs for those aged 65 years and over were not included under the assumption that 65 is the age at which people stop working. This approach is not reflective of the continuing participation of older adults in the workforce, where the proportion of adults aged 65 and over continues to grow. The human capital approach uses lost past and future earnings and household work as a lower bound on the value of a human life. Because of labor market distortions and this article's omission of earnings after age 64, it undervalues women and minorities and places minimal value on the injury and death of people age 65 and older.

The violence prevention and intervention programming costs were restricted to costs internally retrieved from the B.C. Ministry of Public Safety and Solicitor General and are not a comprehensive picture of the existing firearms programming landscape. City-funded programs or funding from other ministries or branches of government were not included.

## Results

There was a total of 108 deaths and 245 hospitalizations resulting from violent firearm injuries in B.C. between 2012 and 2016. The incidence counts for injuries, deaths, offense cases, and offenders during this time period are presented in Table 3. The costs of firearm injuries and deaths in B.C. are detailed in Tables 4, 5.

**Table 3:** Incidence of firearm violent crime deaths, injuries, offense cases, and offenders, B.C. 2012–2016.

**Cost category**	**Violent crime 5-year count**
Deaths	108
Hospitalizations	245
Emergency department visits	164
Disability from hospitalization^i^	56
Police incidents	4,883
Court, prosecution and legal aid offense cases	4,590
Correctional services	1,590
Federal custody offenders	297
Provincial custody offenders	260
Conditional sentences offenders	153
Probation offenders	864
Fine offenders	16
Canada border services agency firearms seized or prosecuted cases	782

i There were 0 disabilities estimated for ED visits.

**Table 4:** Overall average and total costs for firearm violent crime in 2016 CAN Dollars, B.C. 2012–2016.

**Cost category**	**Average cost per count^∧^**	**Average cost per year**	**Violent crime 5-year costs**
**Human costs** ^i^			
Health Care Costs^ii^	NA^*^	$3,910,317	$19,551,584
Productivity Losses			
Workforce^iii^	NA^*^	$17,299,054	$86,495,270
Household	NA^*^	$4,559,470	$22,797,349
Loss of Life	$7,530,000	$162,648,000	$813,240,000
**Total human costs**		**$188,416,841**	**$942,084,203**
**Criminal justice system costs**			
Police^iv^	$69,307	$67,684,862	$338,424,311
Court	$452	$415,258	$2,076,292
Prosecution	$2,993	$2,747,252	$13,736,259
Legal aid	$2,003	$1,838,564	$9,192,819
Correctional services	$63,495	$20,191,252	$100,956,259
Canada border services agency^v^	$77,647	$15,179,946	$60,719,783
**Total criminal justice system costs**		**$105,021,145**	**$525,105,724**
**Violence prevention & intervention programming costs** ^vi^	NA^*^	**$941,000**	**$3,764,000**
**Total costs**		**$294,378,985**	**$1,470,953,927**

i Human costs are based on injuries and deaths from violence, which include homicide, injuries inflicted by another person with intent to injure or kill, those due to legal intervention, and those that are undetermined.

ii Formal caregiving costs are included in the total health care costs. Caregiving is defined as care received by the injured individual within the year of injury and the main condition for which the injured individual sought help. Formal caregiving, which is considered a direct cost, is provided by paid workers and organizations.

iii Informal caregiving provided by family, friends, and neighbors is considered an indirect cost. A proportion of informal caregiving for injury was applied to the workforce productivity losses. Productivity losses are based on deaths, hospitalizations, and disabilities. It is assumed that those seen in emergency departments do not incur any productivity losses.

iv Firearm incidents reported by B.C. police for crimes against the person. Includes offenses where a firearm was present but was not serious.

v Number of national CBSA cases in 2012 was significantly higher than all other years before and after for which data are available with no cause reported, therefore, the number for 2012 was excluded from the average calculations to prevent undue bias. In addition, the expenditure numbers are national, rather than limited to the Pacific Region District. Therefore, there is a likelihood that total costs based on these calculations may be greater than total costs for the Pacific Region District numbers.

vi Gun/Gang violence programming costs based on fiscal years 2013/14-2016/17. Programming costs for later years have increased, however only the costs for the study period have been included.

∧ Count denominators available in Table 3.

* Average costs per incident is not applicable for health care costs, productive losses, and programming costs. Average costs per incident are available for health care subgroups as outlined in Table 5. Productivity losses are presented as an aggregate of all outcomes. Programming costs are at the community level.

**Table 5:** Breakdown of average and total costs for health care, productivity losses and correctional services in 2016 CAN Dollars, B.C. 2012–2016.

**Cost category**	**Average cost per count^∧^**	**Average cost per year**	**Violent crime 5-year costs**
**Health care costs** ^i^			
**Death**	**$11,363**	**$245,445**	**$1,227,223**
**Hospital**	**$55,390**	**$2,714,100**	**$13,570,500**
Hospital care		$1,347,121	$6,735,605
Physician service		$211,580	$1,057,899
Transportation *via* ambulance		$26,016	$130,082
Long-term medical		$1,042,526	$5,212,628
Long-term rehabilitation		$86,857	$434,287
**Emergency department**	**$5,572**	**$182,774**	**$913,869**
Physician service		$11,229	$56,144
Transportation *via* ambulance		$17,344	$86,719
Long-term medical		$145,379	$726,896
Long-term rehabilitation		$8,822	$44,109
**Disability from hospitalization**	**$68,501**	**$767,217**	**$3,836,083**
**Total health care costs**		**$3,910,317**	**$19,551,584**
**Workforce productivity losses** ^ii^			
Death	$770,375	$16,640,094	$83,200,470
Hospital	$931	$45,599	$227,997
Disability from hospitalization	$47,539	$532,436	$2,662,178
**Total workforce productivity losses**		**$17,299,054**	**$86,495,270**
**Correctional services**			
Federal custody^iii^	$293,427	$17,429,560	$87,147,799
Provincial custody^v^	$4,267	$221,895	$1,109,477
Conditional sentences^v^	$19,020	$582,001	$2,910,005
Probation^vi^	$11,341	$1,959,792	$9,798,958
Fines^vii^	-$624	-$1,996	-$9,980
**Total correctional services costs**	**$63,495**	**$20,191,252**	**$100,956,259**

i Formal caregiving costs are included in the total health care costs. Caregiving is defined as care received by the injured individual within the year of injury and the main condition for which the injured individual sought help. Formal caregiving, which is considered a direct cost, is provided by paid workers and organizations.

ii Informal caregiving provided by family, friends, and neighbors is considered an indirect cost. A proportion of informal caregiving for injury was applied to the workforce productivity losses. Productivity losses are based on deaths, hospitalizations, and disabilities. It is assumed that those seen in emergency departments do not incur any productivity losses.

iii Number of offenders admitted to federal custody in B.C. for a crime against the person for a criminal offense where a firearm was present.

^iv^Number of offenders admitted to provincial custody in B.C. for a criminal offense where a firearm was present.

v Number of offenders receiving a conditional sentence in B.C. for a criminal offense where a firearm was present.

vi Number of offenders receiving probation in B.C. for a criminal offense where a firearm was present. Probation cost and fines have been adjusted for inflation in the report but police costs were not as the direct expense amounts were used for each year. The total costs therefore include a combination of inflation-adjusted and non-adjusted costs.

vii Number of offenders receiving a fine sentence in B.C. for a criminal offense where a firearm was present.

∧ Count denominators available in Table 3.

The total estimated cost of all violent firearm injuries and deaths averaged $294,378,985 per year, with a per capita cost of $67 for an average population of 4,404,917; human costs averaged $188,416,841 per year, where health care costs averaged $3,910,317 per year, productivity losses from workforce and household averaged $17,299,054 and $4,559,470 per year, respectively, and loss of life averaged $162,648,000 (Table 4). Hospitalization costs were highest within health care, accounting 69% of health care costs and averaging $2,714,100 per year (Table 5). Criminal justice system costs averaged $105,021,145 per year; policing costs comprised of 64% of the total criminal justice system costs and averaged $67,684,862 per year (Table 4). The cost of firearm violent crime in B.C. per police incident was $301,309, based on an average of 977 incidents per year between 2012 and 2016.

## Discussion

This study clearly demonstrates the significant cost of firearm injury in B.C. and the impacts on the health care and criminal justice systems, and to society at large. This is particularly noted within the criminal justice system, where costs were significantly higher than within the health care system. These *conservative*^1^ estimates indicate that firearms not only pose a threat to public safety, but also a threat to public resources and the broader B.C. economy. The consequences of violent firearm injuries resulted in average costs to society of over $294 million per year, underscoring the value to individuals, families, and society of efforts to prevent firearm violent crime.

There have been a number of studies conducted in other jurisdictions that have identified the costs of firearm injuries (14, 15, 38–43), most of which were conducted in the US and focused primarily on health care costs such as hospitalization and medical costs (14, 38, 40, 41, 43). By comparison, there are a very limited number of firearm injury cost studies that have been conducted in Canada (16, 39). Zhang and Qin (16) investigated the economic and social impacts of firearm crime in Canada and reported total costs of over $3.1 billion per year with a per capita cost of $93. Approximately 80% of these costs were attributed to intangible and third-party costs. Including the costs that were similar to those in our study, the per capita cost in the Canadian study is $63, when the increased costs due to inflation are considered, which is close to our study finding of $67, indicating that firearm-related costs in B.C. are similar to the rest of Canada.

Similar to the findings of Zhang and Qin (16), who reported that $302 million in criminal justice system costs were accounted for by policing services costs (69.5%), corrections (29.7%), courts and prosecution (0.6%), and legal aid (0.2%), this study determined that these proportionate expenditures were 64% for policing services, 19% corrections, 3% courts and prosecution, and 1.8% legal aid costs. In an earlier study by Miller and Cohen (39), the total cost of gunshot wounds in Canada was estimated to be $6.6 billion per year ($235 per capita), including costs for medical care, mental health services, police investigations, productivity losses, funeral expenses, pain and suffering, as well as lost quality of life.

Effective action to prevent firearm incidents can alleviate the human and economic costs that British Columbians currently bear. The B.C. government is addressing firearm violence through a new firearm violence prevention act that was received in 2021 and is anticipated to be regulated in 2022 (44). This act is targeted to regulate the use of firearms, establish new offenses for the unsafe use of firearms as well as prohibit use of firearms in designated places. It is known that with deliberate investment and intervention, rates of firearm violence can decrease. However, these investments must be supported by evidence and closely monitored to ensure anticipated impacts are achieved. The firearm environment is fluid and fast-changing. Initiatives aimed at preventing or mitigating firearm injury must be equally malleable. By ensuring appropriate oversight to these investments and assessing their impacts through research findings provided by this study and others, stakeholders will be better situated to respond to emerging threats in a coordinated and effective manner. These efforts, working together with other broad reaching social support programs (e.g., mental health, housing, food security, income support), have the potential to produce a positive return on investment for society, and more importantly, to save lives (45).

In specifying the multiple cost centers related to firearm injury and death in B.C., this study highlights the scope of opportunities available to policy makers and government for investment in prevention programs to reduce the rate of the firearm injuries and deaths, and decrease costs. Previous research indicates that almost all injuries and injury-related deaths can be prevented (46), and investment in evidence-based prevention policies, programs and initiatives makes a difference. Other primary injury prevention programming has demonstrated significant average return on investment, ranging between $15 and $64 for each dollar invested in initiatives such as the implementation of poison control centers to bicycle helmets and child safety seats (47).

While the prevention of firearm violence may represent a *wicked problem*—a social/cultural issue difficult to solve—(48), prevention programs are demonstrating success. Cure Violence Global, originated in Chicago, is addressing the issue across the US and in 15 other countries *via* strong community partnerships, collaboration with public health and health care systems, and an approach encompassing three core and two implementing components (49). It has produced dramatic results: 56% reduction in killings in Baltimore; 63% reduction in shootings in the South Bronx; and 100% reduction in retaliatory homicides in five of eight Chicago communities. According to Cure Violence Global program calculations, the program is not only saving lives, it is also reducing medical and criminal costs by nearly $16 for each dollar invested (49). The Federal Government of Canada is addressing firearm violence through strengthened firearm laws, including licensing, enhanced record keeping, expanded background checks, and authorization for transportation (50). It is also working to limit access to assault-style firearms, prevent firearms from being smuggled into Canada *via* the Canadian Border Services Agency, and is addressing firearm and gang violence at the provincial/territorial level.

## Conclusions

As the complexity of policing and public health issues increases over time, it is anticipated that the costs associated with firearm injury will continue to rise in parallel. Police and emergency responders continue to experience heightened demands for service and are often inadequately equipped or resourced to respond to these demands. This is particularly evident for cases that require significant investigative resources, man hours, and specialized expertise. In order to effectively respond to and prevent these incidents, it is critical that initiatives to combat firearm violence and injury are appropriately funded, and grounded in evidence-based approaches.

## Data availability statement

The original contributions presented in the study are included in the article/Supplementary material, further inquiries can be directed to the corresponding author.

## Author contributions

Conceptualization: VC. Data curation: FR, TN, and EK. Writing—original draft preparation and visualization: FR. Writing—review and editing: FR, KT, AZ, NP, TN, EK, VC, and IP. Supervision: IP. All authors have read and agreed to the published version of the manuscript.

## Footnotes

^1^Estimates do not include numerous intangible costs associated with firearm injury.
