# Corrigendum: The cost of firearm violent crime in British Columbia, Canada

**DOI:** 10.3389/fpubh.2023.1180968

**Published:** 2023-05-30

**Authors:** Fahra Rajabali, Kate Turcotte, Alex Zheng, Nick Pauls, Tony Nguyen, Evelyn Kalman, Vedrana Covic, Ian Pike

**Affiliations:** ^1^BC Injury Research and Prevention Unit, BC Children's Hospital, Vancouver, BC, Canada; ^2^Public Safety and Police Services Unit, Policing and Security Branch, Ministry of Public Safety and Solicitor General, Victoria, BC, Canada; ^3^Department of Pediatrics, University of British Columbia, Vancouver, BC, Canada

**Keywords:** firearm injury, costs, criminal justice system costs, health care costs, violent crime

In the published article, an error was made in identifying the relevant countries.

A correction has been made to the **Introduction**, paragraph 1. This sentence previously stated:

“Canada ranks 9 out of 36 countries”

This should have been written as:

“Canada ranks 9 out of 36 peer Organization for Economic Co-operation and Development countries”

In the published article, an error was made in providing the average human costs per year.

A correction has been made to **Results**, paragraph 2. This sentence previously stated:

“human costs averaged $183,448,841 per year,”

This should have been written as:

“human costs averaged $188,416,841 per year,”

The authors apologize for this error and state that this does not change the scientific conclusions of the article in any way. The original article has been updated.

