# Supplementary material for: The cost of firearm violent crime in British Columbia, Canada
Source: Front Public Health. 2023 Jan 13;10:938091. doi: 10.3389/fpubh.2022.938091 (PMC9880035; doi:10.3389/fpubh.2022.938091)
Supplement: Supplementary file 1 [file Table_1.pdf]

Supplementary material Data description and sources for criminal justice system costs.

Category	Description	Data Source for Cost Information
**Police**		
Number of firearm incidents	Firearm incidents reported by B.C. Police for crimes against the person.	Uniform crime-reporting incident-based survey, Canadian Centre for Justice Statistics [30]
Police expenditures	Salaries and wages, benefits, and other operating expenses such as accommodation costs, fuel, and maintenance, etc. Note: capital expenditures, funding from external sources, revenues and recoveries are not included.	Statistics Canada [31]
Expenditures on crime-related activities	Canadian police assumed to spend 65% of time on crime-related activities. Other duties include: traffic regulations, offering youth education seminars, coordinating community efforts, patrolling, responding to phone calls (e.g., noise complaints, non-crime emergency calls for help.	Statistics Canada [31]
Severity weights	Weights indicating seriousness of crimes with serious crimes assigned higher weights. Level of seriousness based on courts sentences from all provinces and territories. Note: more serious offences assumed use more police resources.	Statistics Canada [31]
**Court services**	Total provincial expenditures for all Courts Services Branch activities, including information regarding court system and court outcome costs resulting from firearms.	Data internally retrieved from the B.C. Courts Services Branch.
**Prosecution**	Total prosecution expenditure costs including total operating expenditures for PSSG Branches.	Ministry of Attorney General, Government of B.C. [32]
**Legal aid**	Provision of assistance to people who are unable to afford legal representation and access to the court system.	Legal Aid B.C. [33]
**Correctional services**		
Federal custody	• Average daily parole and average daily incarceration costs are used to estimate costs associated with federal custody.<br>• Average custody sentence lengths are calculated using sentencing data and adjusted according to parole grant rates, statutory release, and other data.<br>• Average daily costs are multiplied against these totals to calculate total federal custody sentence costs.	Uniform Crime Report data, Real Time Remote Access (RTRA) program and Integrated Criminal Court Survey data, Statistics Canada.<br><br>Corrections and Conditional Release Statistical Overview jointly published by Public Safety Canada, Correctional Service Canada, the Parole Board of Canada

Category	Description	Data Source for Cost Information
Provincial custody	Offenders sentenced to provincial custody generally are paroled after serving 1/3 of their sentence.[1]<br>• Average daily community supervision and average daily incarceration costs are used to estimate costs associated with provincial custody.<br>• Average custody sentence lengths are adjusted for according to parole grant rates, statutory release, and other data in order to arrive at the total number of days incarcerated, on parole, or on statutory release.<br>• Average daily costs are multiplied against these totals to calculate total provincial custody sentence costs.	The number of offenders admitted to provincial custody was retrieved internally from B.C. Corrections.<br>Provincial full parole grant and failure rate: Parole Board of Canada [34]
Conditional sentence	Sentence where the offender serves in the community, instead of in jail.<br>Numbers of firearm-related offenders receiving a conditional sentence broken down by sex, average daily costs, and average sentence length and multiplied together to calculate total conditional sentence costs for firearm-related offences.	The John Howard Society of Ontario, Fact Sheet, Reconsidering Community Corrections in Ontario [35]
Probation costs	Sentence that requires the offender to follow certain conditions for a set period of time.<br>Numbers of firearm-related offenders receiving probation broken down by sex, average daily costs, and average sentence length were multiplied together to calculate total probation costs for firearm-related offences.	Data for the number of offenders receiving probation were internally retrieved from B.C. Corrections. In 2008, Statistics Canada assumed that the daily probation cost is $20, considering that the seriousness of probation is lower than conditional sentence. Numbers have been adjusted for inflation.
Fines	When the offender is sentenced to pay a fine as a stand-alone sentence or as part of a community sentence.<br>Numbers of firearm-related offenders receiving a fine broken down by sex and average fine amount were multiplied together to calculate total probation costs for firearm-related offences.	Data for the number of offenders receiving a fine as sentence were internally retrieved from B.C. Corrections.<br>Statistics Canada recorded that the average fine amount for these types of offences was $575. Figures between 2012 and 2016 use the same data but account for inflation.

[1] Provincial supervision of offenders on statutory release is not considered here as there is no data available and it is not as strict as the supervision for federal offenders on statutory release

Category	Description	Data Source for Cost Information
**Canadian Border Services Agency criminal investigation**		
National criminal investigation expenditures	CBSA investigators conduct criminal investigations into suspected cases of evasion or fraud with respect to various pieces of border legislation that regulate the importation and exportation of goods, or the admissibility of persons to Canada.	Canada Border Services Agency, Government of Canada [36]
Number of CBSA criminal investigation cases		Canada Border Services Agency, Government of Canada [37]
Number of seizures, prosecutions and cases involving firearms		Data internally retrieved from Canada Border Services Agency
